# Investigation of Interference Models for RFID Systems †

**DOI:** 10.3390/s16020199

**Published:** 2016-02-04

**Authors:** Linchao Zhang, Renato Ferrero, Filippo Gandino, Maurizio Rebaudengo

**Affiliations:** 1 China Academy of Electronics and Information Technology, Shuangyuan Road 11, Shijingshan District, Beijing 100041, China; hune213@163.com; 2 Dipartimento di Automatica e Informatica, Politecnico di Torino, Corso Duca Degli Abruzzi 24, Torino 10129, Italy; filippo.gandino@polito.it (F.G.); maurizio.rebaudengo@polito.it (M.R.)

**Keywords:** RFID, interference model, reader-to-reader collision

## Abstract

The reader-to-reader collision in an RFID system is a challenging problem for communications technology. In order to model the interference between RFID readers, different interference models have been proposed, mainly based on two approaches: single and additive interference. The former only considers the interference from one reader within a certain range, whereas the latter takes into account the sum of all of the simultaneous interferences in order to emulate a more realistic behavior. Although the difference between the two approaches has been theoretically analyzed in previous research, their effects on the estimated performance of the reader-to-reader anti-collision protocols have not yet been investigated. In this paper, the influence of the interference model on the anti-collision protocols is studied by simulating a representative state-of-the-art protocol. The results presented in this paper highlight that the use of additive models, although more computationally intensive, is mandatory to improve the performance of anti-collision protocols.

## 1. Introduction

Radio frequency identification (RFID) is currently a leading technology in the automatic identification and data capture area. With the growing interests of end users in different applications, RFID systems have been exploited in various areas, including smart shopping [1,2], agri-food traceability [3,4], supply chain management [5], construction industry [6], healthcare [7] and temperature tracking for food [8]. Generally, an RFID system is able to increase the process efficiency, by achieving complete traceability and better quality control at a low cost.

A basic RFID system is composed of several tags, one or more readers and a central server. The tag contains data that can be retrieved by a reader located nearby. The server receives, processes and stores the data sent by the readers. Since wireless communication is adopted in an RFID system and the majority of RFID-based applications require a dense reader deployment, data transmissions are susceptible to suffer from interference due to the signals simultaneously broadcast by other readers and tags. The interference in RFID systems can be classified into three types: tag-to-tag, reader-to-tag and reader-to-reader interference [9,10]. Tag-to-tag interference occurs when a reader interrogates multiple tags at the same time. Since this issue affects the performance of all of the RFID systems that involve more than one tag, many approaches have been proposed to address the tag-to-tag collision [11]. Reader-to-tag interference occurs when two or more readers interrogate the same tag simultaneously; this can be solved by separating the interrogation range of the readers. Reader-to-reader interference happens when a reader that is interrogating a tag receives strong signals from one or more readers operating at the same radio frequency, disturbing the weak signal backscattered from the target tag.

So far, although a number of research works related to analyzing reader-to-reader interference have been conducted, there is not an agreed interference model that can address the reader-to-reader collision problem efficiently and accurately. The existing interference models can be roughly categorized into two groups: single interference and additive interference models. In a single interference model, only the collisions between two readers are evaluated. On the contrary, also the interferences caused by a group of readers are considered in an additive interference model: although each reader in the group cannot disturb a certain reader *r* if considered alone, the interference generated by the whole group would prevent *r* from identifying tags.

In this paper, the single and additive interference models are evaluated and compared by simulating an RFID system. On the one hand, the single interference models can be simulated more easily, so they are preferred for a faster evaluation. On the other hand, the additive interference models consider a wider set of possible collisions, in a more coherent way with the real behavior of an RFID network. The goal of the paper is to evaluate if the difference in accuracy between the models are so relevant to recommend the use of an additive interference model for a realistic simulation, despite its computational overhead. In order to emulate the behavior of different RFID applications, three scenarios have been analyzed, and a wide range of system configurations has been considered for each of them. The first scenario consists of a set of independent readers: each reader periodically interrogates the tags with a given probability, and its activity is not affected by the other readers. Without any regulation among the readers, the reader-to-reader interference is more likely to occur, thus preventing the tag identification. In order to increase the performance of the RFID network, the adoption of a channel access method is suggested. Time division multiple access (TDMA) [12] is a well-known channel access scheme for shared medium networks. In a TDMA anti-collision protocol, the readers are scheduled to operate at different time slots. The second scenario considers a basic implementation of the TDMA scheme, with a random choice of the time slot for every reader. Many TDMA protocols have been specifically designed for RFID systems. In particular, the third scenario considers that the readers are scheduled according to the distributed color selection protocol (DCS) [13], which is an *ad hoc* TDMA protocol for RFID networks. The study of the behavior of the RFID system with the three proposed interrogation scenarios allows one to identify two main issues. Firstly, without applying any anti-collision protocol, the reader-to-reader interference dramatically reduces the system performance, *i.e.*, the throughput and the interrogation efficiency. Secondly, the interference model used to simulate a protocol notably affects the estimated performance. Therefore, a precise evaluation of the reader-to-reader anti-collision protocols requires a proper selection of the interference model: in this way, the configuration parameters can be set more suitably in order to obtain a better performance.

The remainder of the paper proceeds as follows. The most common interference models are reviewed in Section 2. Section 3 describes the evaluation parameters. The simulation results under the three scenarios are presented in Section 4, Section 5 and Section 6. Finally, Section 7 is devoted to the conclusions.

## 2. Related Work

This section reviews the most common models that are applied to describe the interference in an RFID network. The models are categorized according to the type of reader-to-reader collision. Collisions that involve only two readers are called direct collisions: they represent the only source of interference assumed by single interference models. Instead, collisions due to multiple readers are called additive collisions and are taken into account by additive interference models. A reader *r* experiences an additive collision if there are no other readers causing a direct collision; however, *r* still cannot identify the tags because other farther readers generate a combined interference.

### 2.1. Single Interference Models

The single interference model represents the direct collisions that occur in the RFID network by means of an interference graph. Each reader is assigned to a node, and two nodes are linked by an edge if the corresponding readers, referred to as neighbors, may collide when they query tags at the same time. The main interference graphs are described in the following.

#### 2.1.1. Bernoulli Random Graph

The Bernoulli random graph is the simplest interference model. Given a set of nodes, the edges are randomly added. The kind of stochastic process applied to build the graph leads to different variants of the Bernoulli random graph. For example, for every pair of nodes, an edge may be added with a probability $p_{Brg}$ independent from every other edge [14]. Another common approach for building a Bernoulli random graph is defined in [15]. The first step is to fix the total number of edges $N_{Brg}$ in the graph. Then, given a set of vertices, all of the possible graphs with $N_{Brg}$ edges are generated. Finally, one member of that set is randomly and uniformly chosen as the Bernoulli random graph.

The Bernoulli random graph models the randomness of the signal propagation in RFID networks due to external causes. Its main advantage is the simplicity of the model, which enables one to exactly express many average properties of the network. However, it does not properly capture the behavior of real-world networks, because the probability of a link between two nodes is independent of their real distance.

#### 2.1.2. Unit Disk Graph

A collision between two RFID readers can be simply predicted by evaluating their distance: the readers may collide if their distance is lower than a threshold *D*. Since the only condition for generating a collision is the relative distance between two readers, two nodes $r_{1}$ and $r_{2}$ are linked by an edge if $r_{1}$ is located within a circle centered in $r_{2}$ and with radius *D*. This interference graph is called the unit disk graph [16].

Some variants of the basic model exist. In a random geometric graph, the position of the readers is randomly chosen inside the unit square ${\left[0,1\right]}^{2}$ [17]. This interference graph becomes a random sector graphs [18] if the antenna of the readers is directional.

Threshold *D* can be evaluated in different ways. An explicit formula is obtained in [19] considering a homogeneous RFID network. In that case, the interrogation range *d*, the transmit power $P_{r}$ and the antenna gain $G_{r}$ are the same for all of the readers. Similarly, the antenna gain $G_{t}$ and the power reflection coefficient $R_{t}$ are the same for all of the tags. Under the condition of a homogeneous network, the value of the threshold is:

(1) $$D=\sqrt[{\alpha }]{\frac{K_{0}\Gamma P_{r}d^{2\alpha }G_{r}^{2}}{R_{t}P_{r}G_{t}^{2}G_{r}^{2}-N_{0}K_{0}^{2}\Gamma d^{2\alpha }}}$$

where *α* is the path loss exponent, Γ is the SINR (signal to interference plus noise ratio) needed to identify the tag, $N_{0}$ is the background noise power and $K_{0}$ is a coefficient that depends on the adopted propagation model (for example, $K_{0}={\left(\frac{4\pi }{\lambda }\right)}^{2}$ in the free space model).

The analysis in [20] determines the presence of a reader-to-reader collision by correlating the distances between the two readers and the group of tags to query. Reader $r_{1}$ can identify tags near it without any interference from reader $r_{2}$ if the following condition is satisfied:

(2) $$|r_{1}-r_{2}\left|\geq \left(1+\Delta \right)\right|r_{1}-t|$$

where Δ is a positive constant, $|r_{1}-r_{2}|$ is the distance between the two readers and $|r_{1}-t|$ is the distance between $r_{1}$ and the farthest tag *t* that is queried. Since the longest distance at which a tag can be identified corresponds to the interrogation range *d*, the longest distance expected in Equation (2) such that two readers experience a reader-to-reader collision is:

(3) D=(1+Δ)d

Equation (3) can be simplified by assigning a constant value to *D* [21], without any explicit correlation to the interrogation range.

#### 2.1.3. Quasi Unit Disk Graph

Ideally, with an omnidirectional antenna, the interference area generated by a reader is circular, because its emitted signals propagate in all directions in the same way. However, this does not hold up in a real scenario, for example due to the irregular shape of the field, some obstacles that obstruct the signal propagation, the atmospheric conditions, *etc*. The quasi unit disk graphs models this situation by introducing a parameter *ρ*, with 0<ρ<1 [22]. As in the unit disk graph, two nodes farther than *D* do not interfere. However, a distance lower than *D* does not entail an interference between the nodes. In detail, in the quasi unit disk graph, two nodes are surely linked only if their distance is lower than ρD. If the distance ranges between ρD and *D*, the existence of the edge is unspecified.

A natural extension of the quasi unit disk graph is the quasi random geometric graph [23], where the nodes are independent and uniformly distributed on the unit square ${\left[0,1\right]}^{2}$. The presence of obstacles that reduce the interference between a pair of readers can be captured by other graphs, such as the faulty random geometric graph [24], which is obtained from a random geometric graph through random removal of edges and vertices.

### 2.2. Additive Interference Models

The criterion followed by the additive interference models for determining the occurrence of a reader-to-reader collision is the evaluation of the SINR at the reader’s antenna. Let be $P_{t,r}$ the power of the reply that reader *r* receives from tag *t* and *I* the interference generated by the other readers. *r* can identify *t* only if the measured SINR is higher than a threshold Γ:

(4) $$\frac{P_{t,r}}{I+N_{0}}\geq \Gamma$$

The contributions to *I* due to each reader can be represented with a weighted undirected graph: the weight $w_{i,0}$ of an edge between two readers $r_{i}$ and $r_{0}$ expresses the interference that the signal emitted by $r_{i}$ provokes on $r_{0}$. Reader $r_{0}$ experiences a reader-to-reader collision if the sum of the weights of all its incoming edges in the graph is so high that Equation (4) is not satisfied:

(5) $$\frac{P_{t,r}}{\sum_{i=1}^{N-1}w_{i,0}+N_{0}}<\Gamma$$

The following subsections present two categories of additive interference models, which differ in the quantity of readers that are considered to compute the interference perceived by the target reader.

#### 2.2.1. Complete Weighted Graph

The most general additive interference models compute the interference received by reader $r_{0}$ by summing the contributions of all of the other readers:

(6) $$I_{0}=\sum_{i=1}^{N-1}P_{r_{i},r_{0}}$$

where *N* is the number of the readers in the network and $P_{r_{i},r_{0}}$ is the power of the signal transmitted by $r_{i}$, measured at $r_{0}$’s antenna. The resulting interference graph corresponds to a complete weighted graph: each node is linked to all of the others, and an edge between two nodes $r_{i}$ and $r_{j}$ has weight $P_{r_{i},r_{j}}$.

Different approaches for estimating the power of the signals $P_{t,r}$ in Equation (4) and $P_{r_{i},r_{j}}$ in Equation (6) exist [19]. A reader can only query tags closer than *d*, so the lowest power of the signal received by a tag is:

(7) $$P_{r,t}=P_{r}\frac{G_{r}G_{t}}{K_{0}d^{\alpha }}$$

Since only a fraction of the signal is backscattered by the tag, the lowest power of the tag’s reply received by the reader is:

(8) $$P_{t,r}=R_{t}P_{r,t}\frac{G_{t}G_{r}}{K_{0}d^{\alpha }}$$

In a homogeneous network, $r_{1}$ generates an interference on the transmission of $r_{0}$ equal to:

(9) $$P_{r_{1},r_{0}}=P_{r}\frac{G_{r}G_{r}}{K_{0}{\left|r_{1}-r_{0}\right|}^{\alpha }}$$

thus, the amount of interference perceived by $r_{0}$ is:

(10) $$I_{0}=\sum_{i=1}^{N-1}P_{r}\frac{G_{r}G_{r}}{K_{0}{\left|r_{i}-r_{0}\right|}^{\alpha }}$$

#### 2.2.2. Random Proximity Graph

The interference caused by a reader on another one is inversely proportional to their distance, as shown in Equation (9), so the greatest amount of interference is due to close readers. Therefore, the total interference can be satisfactorily approximated by summing the contributions of the *k* closest readers. The obtained interference graph is called the random proximity graph [25]. In this graph, the deployment area is the unit square ${\left[0,1\right]}^{2}$, and the nodes are randomly located with a uniform distribution. Each node is linked with its *k* closest nodes, which are identified according to the Euclidean distance.

The random proximity graph can be generalized to a K-nearest neighbor graph [26] if a metric space other than the Euclidean space is considered. In that case, the number of dimensions is higher than two, and a measure of distance different from the Euclidean distance may be applied.

## 3. Simulation Setup

An *ad hoc* simulator for RFID networks [27] has been adopted to test and compare the single and additive interference models. In particular, the unit disk graph and the complete weighted graph models are chosen as representative of the two classes of models.

In the experiments, the deployment area is a 1000 m × 1000 m field. In order to evaluate the influence of the reader density, the number of readers varies from 10 to 50. The RFID network is considered homogeneous. Table 1 shows the values of the parameters adopted in the simulation, in accordance with [28]. In particular, *D* is computed by substituting the adopted values into Equation (1). *D* is used to determine whether two readers are neighbors in the unit disk graph model.

The following metrics have been adopted to compare the interference models:

- success rate: the ratio of the number of successful interrogations to the amount of attempts;
- throughput: the average number of successful interrogations performed per reader during a specific amount of time;
- percentage of additive collisions (for the additive interference model only): the ratio of the number of collisions caused by more than one reader to the amount of reader-to-reader collisions.

**Table 1:** Parameters of the interference models.

Parameter	Symbol	Value
Interrogation range	*d*	5 m
Reader antenna gain	$G_{r}$	6 dBi
Tag antenna gain	$G_{t}$	1 dBi
Tag’s power reflection coefficient	$R_{t}$	3/4
Reader’s transmit power	$P_{r}$	30 dBm
Model coefficient	$K_{0}$	1/$G_{r}^{2}$
Path loss exponent	*α*	2
SINR Threshold	Γ	10
Noise power	$N_{0}$	0
Threshold distance	*D*	288.68 m

As the activity of the readers depends on the application requirements, different behaviors are tested by considering three scenarios in the simulations:

- probabilistic interrogation: at every time slot, each reader has a probability *p* of querying tags;
- slotted interrogation: the communication is organized in rounds composed by a fixed number of time slots, and each reader randomly chooses a time slot to interrogate the tags;
- DCS protocol: the readers are scheduled according to a state-of-the-art TDMA protocol [13].

Different configuration parameters have been considered for every scenario: each case is simulated 100 times in order to reduce the effect of randomness.

## 4. Probabilistic Interrogation Scenario

In the current scenario, the activity of the readers is modeled as a Bernoulli process, *i.e*., a discrete-time stochastic process. The simulation time is divided into iterations of the same length: in the experiments, each simulation consists of 2000 iterations. The last instant of an iteration is called the interrogation point. The readers are allowed to interrogate the tags only at the interrogation points, with a probability *p*. The probability *p*, which represents the interrogation frequency of the system, is selected according to the goal of the system: the higher *p*, the more often the tags are queried. The activity of a reader at the interrogation point is independent of the other readers. Furthermore, the operation is memoryless, *i.e*., the behavior of a reader is independent of the previous iterations.

The throughput of the readers depends on *p* and on the density of the nodes: for example, few tag identifications are expected to be completed with a low density and a low value of *p* (because few interrogations are attempted) or with a high density and a high value of *p* (because there is too much interference generated by the simultaneous interrogations). Different simulations of the probabilistic interrogation scenario are performed, with *p* ranging from 0.1 to 1.

### 4.1. Success Rate in the Probabilistic Interrogation Scenario

**Figure 1:**
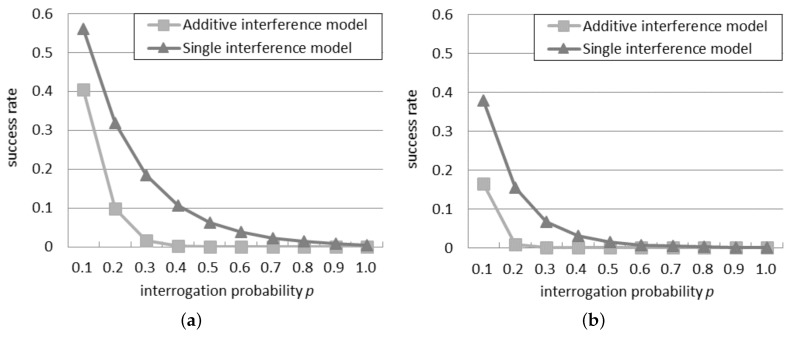
Success rate in the probabilistic interrogation scenario: (**a**) 30 readers; (**b**) 50 readers.

Figure 1 compares the success rate in the two models, with 30 or 50 readers in the network. It can be observed that the success rate is always lower than 0.6 for 30 readers and 0.4 for 50 readers: this indicates that an anti-collision algorithm is necessary to reach a good probability of tag identification in an RFID system. Beyond a certain threshold of *p*, called bottleneck probability, it is impossible to perform any tag identification because the quantity of readers simultaneously querying is too high. As shown in Figure 1a, with 30 readers, the bottleneck probability is 0.8 according to the single interference model, but it falls to 0.4 according to the additive interference model. Similarly, as shown in Figure 1b, with 50 readers, the bottleneck probability reduces from 0.6 to 0.2, by passing from the single to the additive interference model. This remarkable difference in the bottleneck probability reveals to what extent the behavior of the readers differs according to the single and additive interference models.

In both the networks, the successful ratio results always in being higher by using the additive interference model than by using the single interference one, since the additive interference model is able to consider a larger number of reader-to-reader collisions with respect to the single interference one.

The success rate in Figure 1b is always lower than the corresponding rate in Figure 1a, because, as the network density grows, the interrogations of a reader influence more neighbors.

### 4.2. Throughput in the Probabilistic Interrogation Scenario

**Figure 2:**
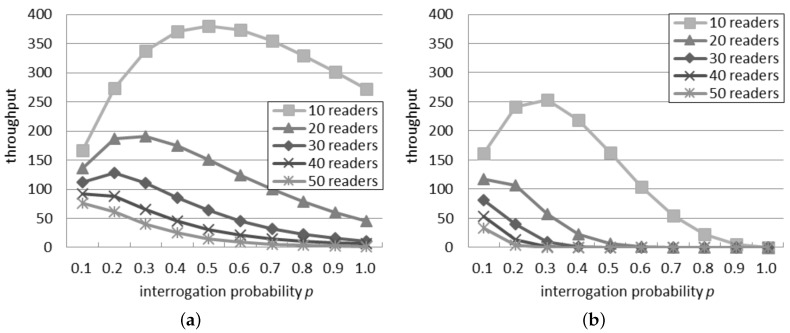
Throughput in the probabilistic interrogation scenario. (**a**) Single interference model; (**b**) Additive interference model.

Figure 2a considers the probabilistic interrogation scenario and shows the throughput computed by using the single interference model. Considering a quantity of readers between 10 and 30, initially, the throughput increases, but after a peak, it decreases. The position of the peak is due to *p*: as the quantity of readers increases, the peak is reached with a lower probability. When there are more than 30 readers, the throughput constantly decreases as *p* grows. For a fixed interrogation probability, the throughput decreases if the quantity of readers grows, because a larger number of readers involves a greater number of simultaneous interferences.

Figure 2b presents the throughput expected by the additive interference model. The peak point is reached with a value of *p* higher than 0.1 only if there are 10 readers or less in the network. In accordance with the single interference model, the throughput decreases if the quantity of readers grows due to the bigger amount of collisions among them. It can be also observed that the throughput quickly becomes zero in some system configurations (e.g., 30 readers with *p* > 0.4), in accordance with the success rate shown in Figure 1.

By comparing Figure 2a,b, it can be noted that the value of *p* associated with the peak point, which represents the best configuration, notably differs according to the two interference models when there are 30 readers or less. Instead, the two models agree with more than 40 readers: in this case, the peak point is always reached with p=0.1.

**Figure 3:**
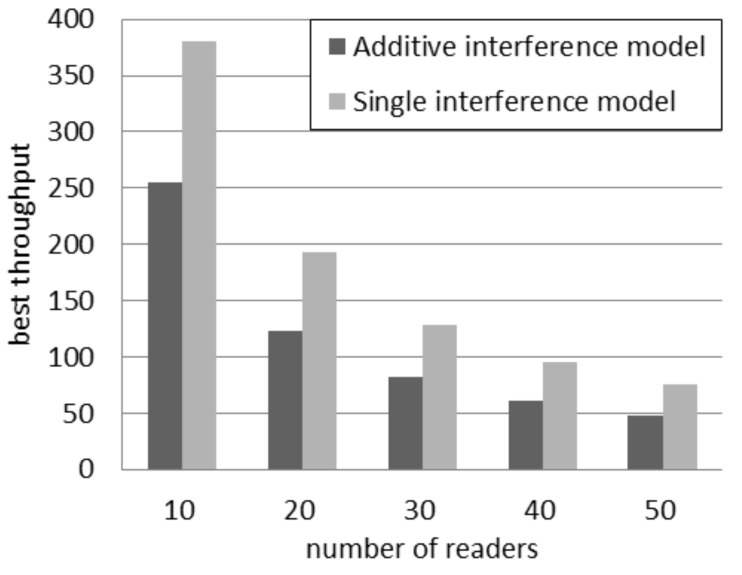
Best throughput in the probabilistic interrogation scenario.

Independently of the number of readers, the highest throughput achievable according to the single interference model is higher than the throughput estimated by using the additive interference one. Figure 3 compares the best throughput according to the two interference models considering a quantity of readers between 10 and 50. It can be observed that the absolute difference in throughput decreases if the quantity of readers grows. However the relative difference increases. In fact, the percentage difference, *i.e*., the difference between the two values divided by their average, rises from 39% with 10 readers to 45% with 50 readers.

### 4.3. Percentage of Additive Collisions in the Probabilistic Interrogation Scenario

**Figure 4:**
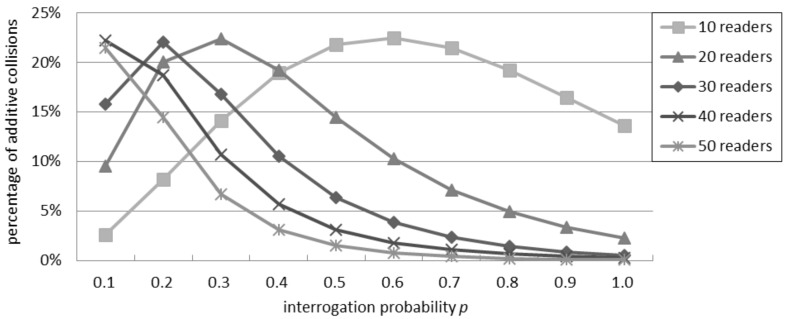
Percentage of additive collisions for the additive interference model in the probabilistic interrogation scenario.

Figure 4 shows the ratio of collisions due to an additive interference. When the number of readers is between 10 and 30, the ratio initially grows as the interrogation probability grows, because the number of collisions is limited and the relative quota of the additive ones is significant. However, after reaching a peak point, the percentage of additive collisions starts to decrease as *p* increases, because the direct collisions become predominant: in this case, it is more likely for a reader to have a neighbor that tries to query the tags nearby, causing a collision. With 40 or more readers, the ratio of additive collisions over the total quantity of collisions decreases with a progressively lower impact on the total interference. Independently of the number of readers, the greatest ratio of additive collisions over the total quantity of collisions is about 22%.

## 5. Slotted Interrogation Scenario

In this scenario, the communications are organized in rounds with the same duration. The rounds are composed by time slots. Each reader randomly selects a time slot to query tags. If there is a collision, the reader randomly picks a new slot for the further rounds. If the quantity of time slots is high, after a self adjustment phase, the system becomes steady, and the readers are able to query tags without reader-to-reader collisions.

This slotted mechanism is at the base of any TDMA protocol, because it reduces the reader-to-reader collisions. Nevertheless, the quantity of slots per round may affect the performance. In fact, a large quantity of time slots does not correspond directly to a high throughput, because if there are too many time slots, some of them could remain unused. In this section, the trade-off between the throughput and the number of collisions is evaluated trough simulations. All of the simulations last 1000 s, and the length of the time slot is set to 0.5 s.

### 5.1. Success Rate in the Slotted Interrogation Scenario

**Figure 5:**
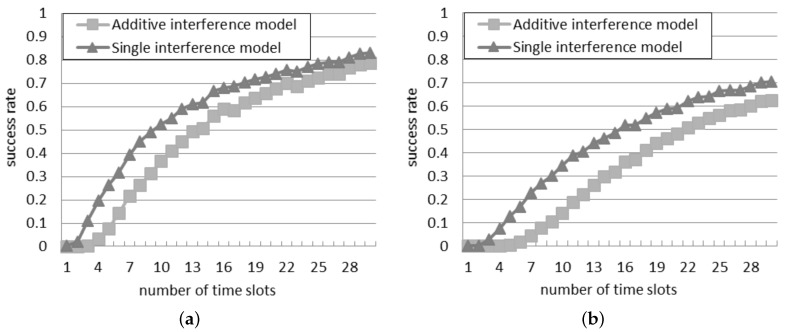
Success rate in the slotted interrogation scenario: (**a**) 30 readers; (**b**) 50 readers.

Figure 5 illustrates the success rate according to the two interference models. The rate monotonically climbs up as the number of time slots increases, because the probability that more than one reader transmits at the same time slot reduces. The highest number of time slots adopted in the simulations is 30. In this case, with 50 readers, the success rate is equal to 0.63 according to the additive interference model, and it is even higher, equal to 0.71, according to the single interference model.

With respect to the results obtained in the probabilistic interrogation, the success rate is remarkable higher, since the interrogations are scheduled in different time slots, and this reduces the reader-to-reader collisions. However, Figure 5a shows that if the quantity of time slots and of readers are the same, the success rate is lower than one. This result is due to the starting unsteady phase, during which the readers look for a free time slot.

Instead, the difference between the two models is primarily influenced by the quantity of slots: it reduces as the quantity of slots increases. With a larger quantity of time slots, the readers have a greater probability to be assigned to a different time slot, with negligible interference among them.

### 5.2. Throughput in the Slotted Interrogation Scenario

**Figure 6:**
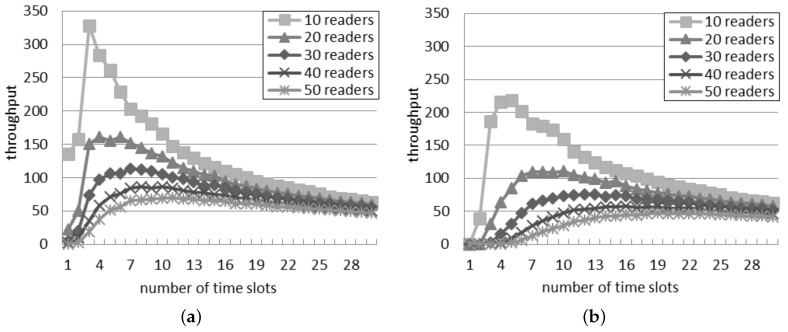
Throughput in the slotted interrogation scenario. (**a**) Single interference model; (**b**) Additive interference model.

Figure 6 shows the throughput estimated by the two interference models. The general behavior is similar in both the interference models and with any network density: the throughput raises to a peak and then slowly decreases. The position of the peak changes according to the number of readers. In both models, a large quantity of readers corresponds to many time slots needed to obtain the best throughput.

The throughput predicted by the single interference model is always larger than the throughput computed with the additive model, while the quantity of slots corresponding to the best throughput is lower for the single interference model (e.g., with 10 readers, five time slots per round correspond to the best throughput in Figure 6b; however, in Figure 6a, the supposed best number of time slots is three). The difference is greater for a large quantity of readers: with 50 readers, the best quantity of slots per round is 11 and 19 for the single and additive interference models, respectively.

**Figure 7:**
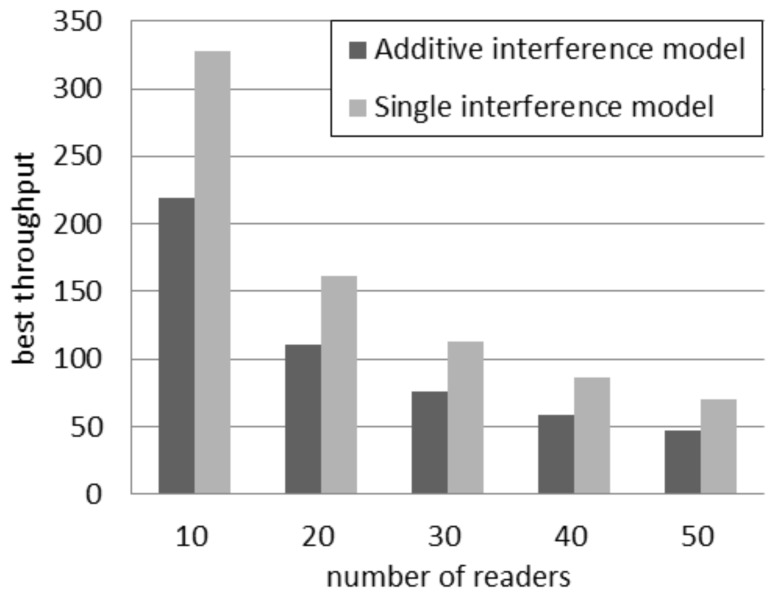
Best throughput in the slotted interrogation scenario.

Figure 7 shows the comparison of the best throughput with the considered models. The percentage difference between the best throughput supposed by the considered models is almost constant and independent of the number of readers. It oscillates in the interval between 37% and 40%. For a larger quantity of readers, the best throughput is achieved by using a higher quantity of slots, as shown in Figure 6. As a consequence, the percentage difference between the best throughput in the two models is not affected by the number of readers, because it is compensated by using a different quantity of slots.

### 5.3. Percentage of Additive Collisions in the Slotted Interrogation Scenario

**Figure 8:**
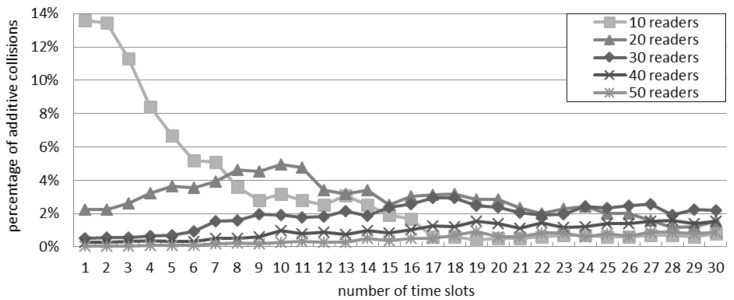
Ratio of additive collisions with the slotted interrogation scenario.

Figure 8 considers the scenario of slotted interrogation and shows the ratio of additive collisions over the total quantity of collisions. Only with 10 readers, the ratio of additive collisions is affected by the number of slots per round. This confirms the trend of Figure 7: by varying the number of readers, the best throughput is achieved by using a different quantity of slots. Since the percentage of additive collisions is poorly affected by the quantity of slots, the percentage difference is similar with both models.

## 6. DCS Scenario

The distributed color selection (DCS) protocol [13] is one of the most popular TDMA anti-collision protocols. The main idea is to use a certain number of colors (maxColors) to distinguish all of the readers in the system, so that each reader has the smallest possible number of neighbors with the same color. Initially, each reader selects a color and queries the tags only during the corresponding time slot. Once a reader detects an interrogation failure, it randomly changes the color and broadcasts the new color to its neighbors: this message is referred to as a kick. Whenever a reader receives a kick with the same color as its own, it selects another color.

With respect to the previous scenario, in DCS, the choice of a new time slot for a reader may be due not only to its own activity (*i.e.*, after an interrogation failure), but also to the information received from its neighbors (*i.e.*, when they send a kick). However, no countermeasures are planned in DCS if a reader experiences an interference generated by a group of other readers. This event is considered only by the additive interference model, so the performance of DCS is expected to change depending on the considered interference model. The goal of this section is to investigate the different performance of the DCS protocol according to the single and additive interference models. The length of each slot is 0.5 s, and the total simulation time is 1000 s.

### 6.1. Success Rate in the DCS Scenario

**Figure 9:**
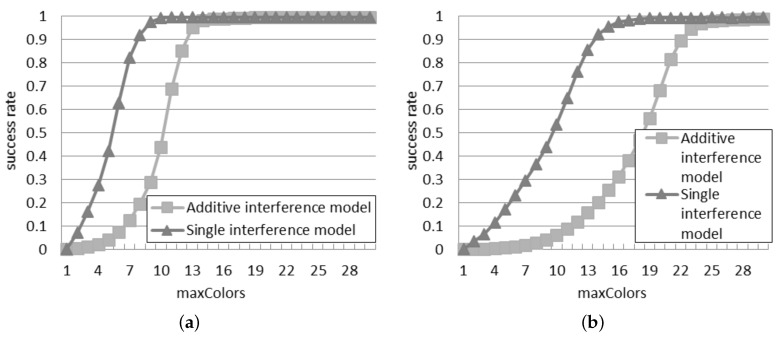
Success rate in the distributed color selection (DCS) scenario: (**a**) 30 readers; (**b**) 50 readers.

Figure 9 presents the success rate achieved with DCS according to the two interference models. The values reported in both models decrease as more readers are deployed, since more collisions appear when the density grows. When there are 30 readers, DCS shows a good performance (*i.e.*, the success rate is larger than 99%) with maxColors≥ 10 in the single interference model and maxColors≥ 15 in the additive interference model. However, when the number of readers grows to 50, a big raise of maxColors is required in order to get the same performance: the values of maxColors required to reach a success rate higher than 99% become 18 and 25 for the single and additive interference models, respectively.

It can be observed that the difference between the two models is much larger than in the case of probabilistic and slotted interrogations. For example, in Figure 9a, if the value of maxColors is lower than or equal to three, the success rate is close to zero according to the additive interference model: this means that three colors are not enough to avoid collisions in such a dense deployment. On the contrary, with three colors, the single interference model supposes that 18% of the interrogations are successful. According to the additive interference model, a similar success rate is achieved only if maxColors is at least equal to eight. Furthermore, the difference between the considered interference models is larger for a greater quantity of readers. In Figure 9b, the additive interference model estimates a success rate close to zero with a value of maxColors lower than or equal to six: this configuration leads to a success rate of 24% in the single interference model. maxColors should be at least equal to 15 to achieve such a success rate in the additive interference model. This analysis reveals that the choice of the interference model plays an important role in the design of an anti-collision protocol, in order to set the proper configuration parameters (like the value of maxColors in DCS depending on the network density).

### 6.2. Throughput in the DCS Scenario

**Figure 10:**
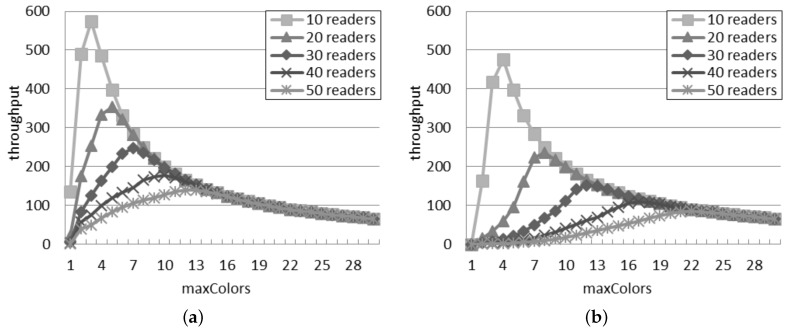
Throughput in the DCS scenario. (**a**) Single interference model; (**b**) Additive interference model.

Figure 10 presents the throughput of the DCS protocol in the single and additive interference models. On the one hand, a large value of maxColors results in a good ability to avoid collisions, but on the other hand, it also means fewer interrogation attempts at the same time. This explains the trend of the throughput in both the inference models: initially, the throughput grows as maxColors increases, because the number of reader-to-reader collisions reduces. However, after reaching a peak, the throughput decreases if maxColors further increases, because the number of reader-to-reader collisions keeps stable, but the readers query tags less frequently.

The single interference model tends to overestimate the performance of the DCS protocol, since it ignores the additive collisions. Besides the throughput loss, the additive collisions may affect the behavior of the network in other ways: first, more kick packets are exchanged in the additive interference model because a reader can collide also with a group of readers and not only with a single one, so it needs to change color more often; secondly, it is more likely that its new color will cause another collision, because, besides the colors of its neighbors, the reader should avoid also the colors of readers that can generate an additive collision.

### 6.3. Best Configuration in the DCS Scenario

As shown in Table 2, the optimal value of maxColors in order to achieve the highest throughput depends on the interference model. For example, in a network with 40 readers, maxColors should be 10 according to the single interference model, but that value turns to 17 according to the additive interference model. The difference between that optimal value grows as the quantity of readers rises: for example, the difference raises from one, with 10 readers, to 10, with 50 readers. Considering that the additive model is more accurate, it can be concluded that the single interference model is not able to compute the optimal parameter setting for the DCS protocol, especially if there are many readers. The fourth and the fifth columns in Table 2 report the throughput according to the additive interference model for two distinct values of maxColors: the value suggested by the single interference model (as shown in the second column) and the value suggested by the additive interference model (as shown in the third column). If there are more than 10 readers, by selecting the value of maxColors according to the single interference model, the throughput loss, with respect to the configuration selected according to the additive interference model, ranges from 59.4% to 67.4%. The difference is less significant with 10 readers because the optimal value of maxColors suggested by the two interference models does not differ significantly.

**Table 2:** Analysis of the optimal value of maxColors in the DCS scenario.

Number of Readers	Optimal maxColors		Throughput in Additive Model		Through-Put Loss
	SingleModel	AdditiveModel	SingleConfiguration	AdditiveConfiguration	
10	3	4	418.84	474.98	11.8%
20	5	8	96.03	236.71	59.4%
30	7	12	49.10	150.81	67.4%
40	10	17	40.85	109.12	62.6%
50	12	22	28.68	85.15	66.3%

### 6.4. Percentage of Additive Collisions in the DCS Scenario

**Figure 11:**
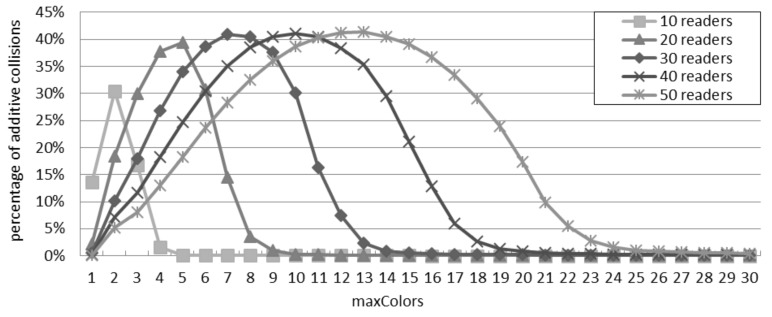
Percentage of additive collisions for the additive interference model in the DCS scenario.

It can be observed from Figure 11 that the influence of additive collisions on the performance initially increases up to a peak and then decreases, with respect to the growing value of maxColors. For example with 40 readers, the percentage of additive collisions firstly grows to the peak point at 41% (with maxColors=10), because the direct collisions deceases as maxColors grows, which is also reflected in Figure 10. Afterwards, the percentage of additive collisions drops down until approaching 0%. In fact, with many time slots, DCS achieves a stable state in which each reader can perform the interrogation without interferences from other readers, since each reader owns a unique time slot.

## 7. Conclusions

This paper investigates the interference models for the reader-to-reader collisions in RFID systems. In order to evaluate the effects of the models on the estimation of the performance of RFID systems, two general cases, namely the probabilistic interrogations and the slotted interrogations, are considered and simulated. The success rate, the throughput and the percentage of additive collisions are taken into account as analysis metrics. Moreover, DCS is implemented and simulated in order to study the influence of the interference models on a state-of-the-art TDMA anti-collision protocol. The goal of the investigation was to find if and when the simpler single interference models can be used instead of the more accurate additive interference models, without compromising the results of the analysis. In general, a single interference model underestimates the impact of the reader interference on the system performance. Furthermore, in the design of an anti-collision protocol, if the configuration parameters are chosen according to a single interference model, the system may not be able to achieve the optimal performance, as supposed by the additive interference model. Therefore, an accurate study on the RFID reader-to-reader collisions should prefer the additive interference model.
